# Yttrium Complexes of Arsine, Arsenide, and Arsinidene Ligands**

**DOI:** 10.1002/anie.201500173

**Published:** 2015-02-05

**Authors:** Thomas Pugh, Andrew Kerridge, Richard A Layfield

**Affiliations:** School of Chemistry, The University of Manchester Oxford Road, Manchester, M13 9PL (UK); Department of Chemistry, Lancaster University Lancaster, LA1 4YB (UK)

**Keywords:** arsenic, arsinidene ligands, lithium, rare-earth elements, yttrium

## Abstract

Deprotonation of the yttrium–arsine complex [Cp′_3_Y{As(H)_2_Mes}] (**1**) (Cp′=η^5^-C_5_H_4_Me, Mes=mesityl) by *n*BuLi produces the μ-arsenide complex [{Cp′_2_Y[μ-As(H)Mes]}_3_] (**2**). Deprotonation of the As–H bonds in **2** by *n*BuLi produces [Li(thf)_4_]_2_[{Cp′_2_Y(μ_3_-AsMes)}_3_Li], [Li(thf)_4_]_2_[**3**], in which the dianion **3** contains the first example of an arsinidene ligand in rare-earth metal chemistry. The molecular structures of the arsine, arsenide, and arsinidene complexes are described, and the yttrium–arsenic bonding is analyzed by density functional theory.

Rare-earth metal compounds containing soft heteroatom donor ligands have attracted considerable interest in recent years.^1^–^6^ The combination of Lewis acidic M^3+^ cations with heavy p-block donor atoms results in a hard–soft mismatch that can lead to unusual bonding properties and to distinct reactivity. Within this context, rare-earth metal complexes of anionic phosphorus donor ligands such as phosphide (R_2_P^−^) have been extensively studied.^7^ A key development occurred in 2008, when a lutetium phosphinidene (RP^2−^) complex was structurally characterized and its phosphinidene transfer reactivity towards aldehydes and ketones demonstrated.^8^ Phosphinidene complexes of other rare-earth metals were subsequently reported, and their phosphinidene transfer chemistry and small-molecule activation reactions described.^9^–^14^ Despite the increased activity in rare-earth metal phosphinidene chemistry, the area is considerably underdeveloped relative to transition metal phosphinidene chemistry.^15^ A terminally bonded phosphinidene ligand remains a key target in rare-earth metal chemistry, although a uranium complex of such a ligand was reported recently.^16^

The chemistry of rare-earth metal complexes with arsenic donor ligands is almost entirely unexplored: arsenide (R_2_As^−^) complexes are rare,^17^–^22^ and arsinidene (RAs^2−^) ligands are unknown in rare-earth metal chemistry. The development of synthetic routes to rare-earth metal arsinidene complexes could lead to more novel reactivity, such as arsinidene transfer, and would also furnish new opportunities for using arsenic ligands to influence the electronic structure and magnetism of lanthanide(III) complexes. With these possibilities in mind, we now report the first example of a rare-earth metal arsinidene complex.

Our strategy involved the initial synthesis of a primary arsine complex of yttrium to establish the metal–arsenic bond, followed by deprotonation of the {YAsH_2_R} unit to give corresponding yttrium–arsenide and yttrium–arsinidene complexes. Thus, adding one stoichiometric equivalent of mesitylarsine to Cp′_3_Y in toluene led to the formation of [Cp′_3_Y{As(H)_2_Mes}] (**1**) (Cp′=η^5^-C_5_H_4_Me, Mes=mesityl), which was crystallized as colorless blocks in 88 % yield (Scheme 1). To obtain the yttrium arsenide complex, **1** was dissolved in toluene and one equivalent of *n*BuLi was added. The ensuing work-up allowed isolation of the trimetallic μ-arsenide complex [{Cp′_2_Y[μ-As(H)Mes]}_3_]⋅toluene (**2**⋅toluene) in 59 % yield.Deprotonation of **2** by *n*BuLi in thf, followed by crystallization from the same solvent, resulted in formation the heterobimetallic yttrium–lithium μ-arsinidene complex [Li(thf)_4_]_2_[{Cp′_2_Y(μ-AsMes)}_3_Li]⋅thf, [Li(thf)_4_]_2_[**3**]⋅thf, as orange crystals in 73 % yield.

**Scheme 1:**
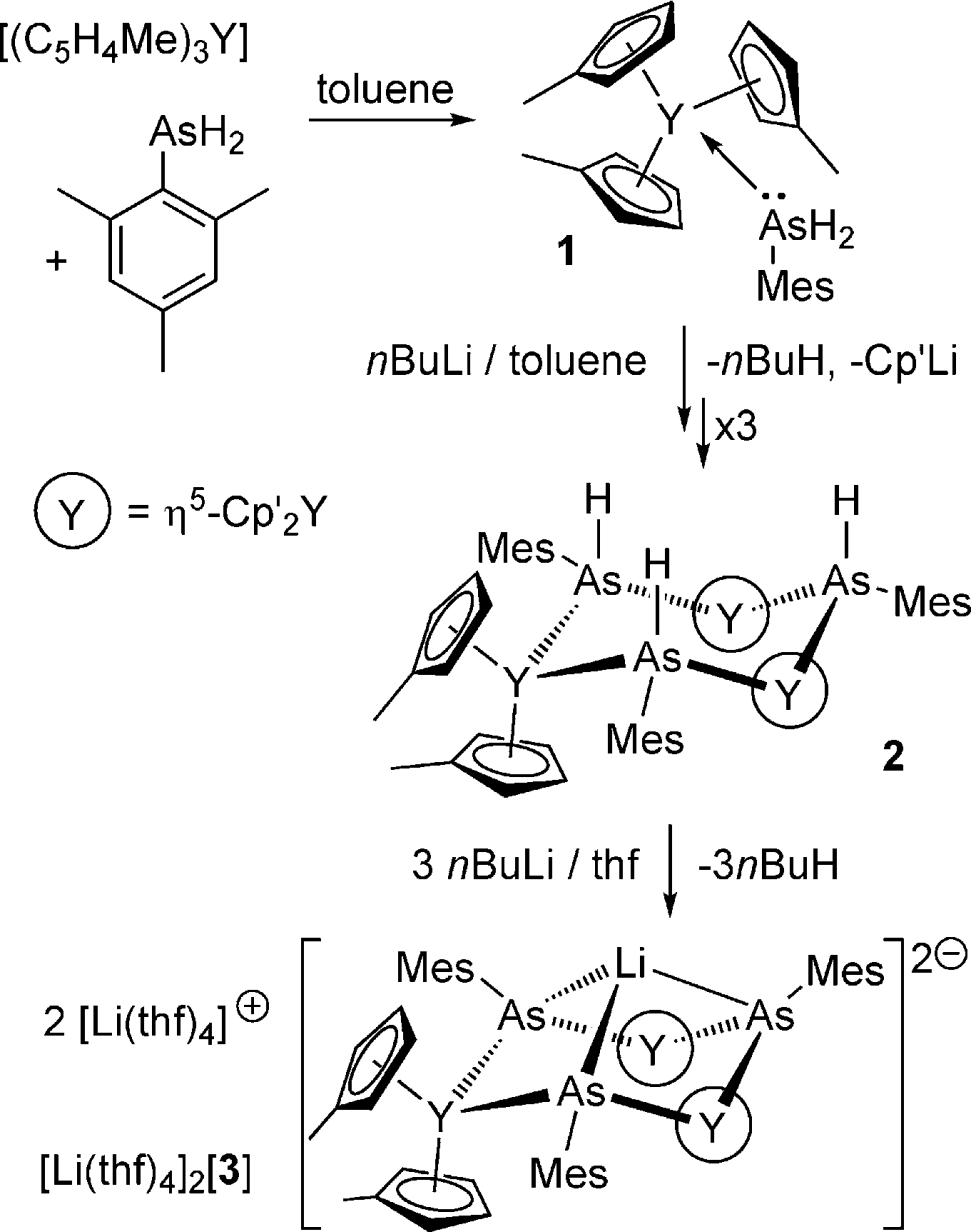
Synthesis of the complexes described herein.

The molecular structure of the yttrium arsine complex **1** (Figure 1) features a Y1–As1 bond of length 3.0945(6) Å, and a Y1-As1-C19 angle of 122.55(7)°. The three Cp′ ligands are η^5^-coordinated to yttrium, with Y–C bond lengths in the range 2.672(3)–2.746(3) Å (average 2.706 Å). The IR spectrum of **1** shows characteristic As–H stretches at 2120 and 2154 cm^−1^ (Supporting Information, Figure S9). The ^1^H NMR spectrum of **1** in [D_6_]benzene (Supporting Information, Figure S2) features a resonance corresponding to the arsine protons at δ(^1^H)=3.12 ppm, the mesityl methyl groups occur at δ(^1^H)=2.08 and 2.18 ppm, and the mesityl aromatic protons occur at δ(^1^H)=6.67 ppm. The Cp′ methyl group occurs at δ(^1^H)=1.95 ppm and the Cp′ CH protons occur at δ(^1^H)=5.86 and 5.77 ppm. The ^1^H NMR spectrum of **1** shows no evidence for free arsine.

**Figure 1:**
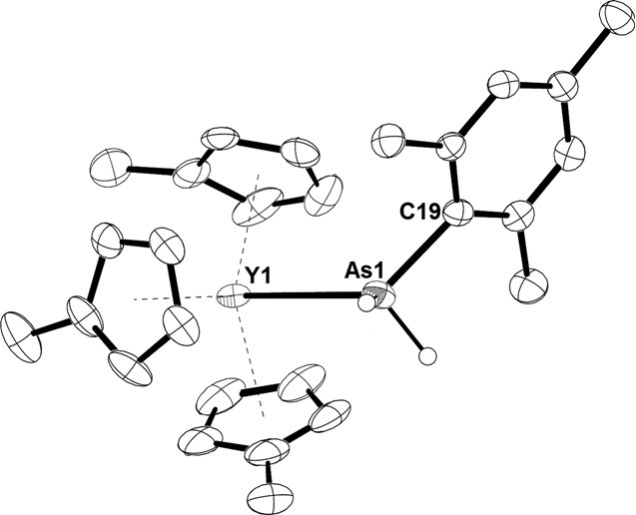
Molecular structure of 1, with ellipsoids set at 50 % probability.^27^ Unlabeled atoms are carbon; hydrogen atoms, except those bonded to arsenic, are not shown.

The only rare-earth metal arsine complex reported to date contains a macrocyclic amidoarsine ligand coordinated to yttrium, with Y–As bond lengths in the range 2.9545(7)–2.9968(7) Å.^17^ Complex **1** is therefore the first rare-earth metal complex of a primary arsine ligand.

The molecular structure of the yttrium arsenide **2** (Figure 2) consists of a central Y_3_As_3_ chair-like ring, with each yttrium ligated by two μ-arsenide ligands and two η^5^-Cp′ ligands. The Y–As bond lengths in **2** are in the range 2.977(2)–3.019(2) Å (average 2.998 Å), and therefore they are, on average, approximately 0.10 Å shorter than the Y–As bond in **1**, which is due to the stronger electrostatic attraction between yttrium and the arsenide ligand. The As-Y-As bond angles are in the range 88.66(5)–96.26(5)°. The Y–C bond lengths in **2** are 2.59(1)–2.67(1) Å, and the average Y–C distance of 2.63 Å is approximately 0.08 Å shorter than in **1**. The Y-As-Y angles in **2** are 130.56(5), 135.09(6), and 135.66(6)°, and each arsenic center carries an *exo* mesityl substituent. The As–H stretching vibrations were observed in the IR spectrum at 2120 and 2154 cm^−1^ (Supporting Information, Figure S9).

**Figure 2:**
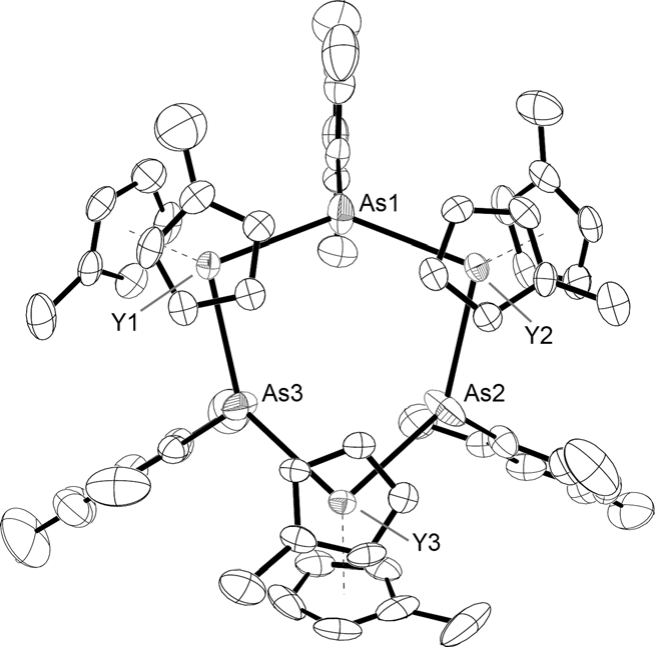
Molecular structure of 2, with ellipsoids set at 50 % probability.^27^ Unlabeled atoms are carbon; hydrogen atoms are not shown.

The ^1^H–^13^C HSQC spectrum of **2**⋅toluene at 298 K reveals that two proton environments at δ(^1^H)=2.51 and 2.60 ppm, with relative integrals of 1:2, do not engage in ^1^*J* coupling to carbon, which identifies them as the As–H protons and indicates that there are two magnetically inequivalent arsenic environments. The ^1^H NMR spectrum of **2**⋅toluene features four resonances in the range δ(^1^H)=6.78–6.94 ppm and 5.84–6.51 ppm, which correspond to the six mesityl CH protons and the 24 Cp′ CH protons, respectively (Supporting Information, Figures S3–S5). The various CH_3_ environments occur in the range δ(^1^H)=1.72–2.66 ppm.

Complex **2** is the first rare-earth metal complex of a primary arsenide ligand; however, several crystallographically characterized rare-earth metal complexes of secondary arsenide ligands have been reported.^18^–^22^ A range of synthetic routes have been employed to access secondary arsenide complexes, including, for example, deprotonation of Ph_2_AsH by the lutetium–lithium methyl complex [Cp_2_Lu(μ-CH_3_)_2_Li(tmeda)] (tmeda=*N*,*N*,*N*′,*N*′-tetramethylethylenediamine), which resulted in the formation of the arsenide-bridged species [Cp_2_Lu(μ-AsPh_2_)_2_Li(tmeda)].^17^ Activation of As–As bonds by samarium(II) reduction has also been used to access arsenide complexes such as [Cp*_2_SmAsPh_2_], which features a terminally bonded [Ph_2_As]^−^ arsenide ligand.^19^, ^20^ Lanthanide(II) arsenide and arsolyl complexes can be accessed by salt metathesis reactions of LnI_2_ with alkali-metal arsenide salts; for example, Mes_2_AsK reacts with SmI_2_ to give *trans*-[(Mes_2_As)_2_Sm(thf)_4_].^21^, ^22^

The structure of the arsinidene-ligated complex dianion **3** (Figure 3) also consists of a central chair-like Y_3_As_3_ core, with three arsinidene ligands bridging the yttrium centers. A lithium cation caps the core of the structure and bonds to the three arsenic donors, such that the arsinidene ligands adopt an overall μ_3_-bridging mode. The Y–As bond distances in **3** are 2.8574(6)–2.8893(7) Å (average 2.8722 Å), making them shorter on average than the Y–As bonds in **2** by more than 0.12 Å. It is also noteworthy that the Y⋅⋅⋅Y separations in **2** are 5.465–5.548 Å, whereas those in **3** are 5.266–5.314 Å; overall, therefore, the Y_3_As_3_ core of **3** is more compact than that of **2**. Relative to **2**, a broader range of Y–C bond lengths, that is, 2.59(2)–2.731(6) Å, and a greater average Y–C bond length of 2.67 Å, are found in **3**. The distortion of the Y_3_As_3_ chair conformation in **3** is reflected in the As-Y-As and Y-As–Y bond angles of 91.59(2)–94.87(2)° and 133.59(2)–136.49(2)°, respectively. The lithium cation in **3** is ligated by three arsinidene ligands and resides 0.889(8) Å above the mean plane of the arsenic atoms. The Li–As bond lengths are 2.539(8), 2.563(7) and 2.615(8) Å, and the As-Li-As angles are 107.2(3), 108.2(3) and 110.0(3)°. An *ortho* methyl group on one mesityl substituent is oriented towards Li1, and the relatively short Li1⋅⋅⋅C54 distance of 2.777(8) Å may indicate an agostic interaction similar to that observed in other lithium complexes containing CH_2_R substituents (R=H, alkyl, silyl).^23^

**Figure 3:**
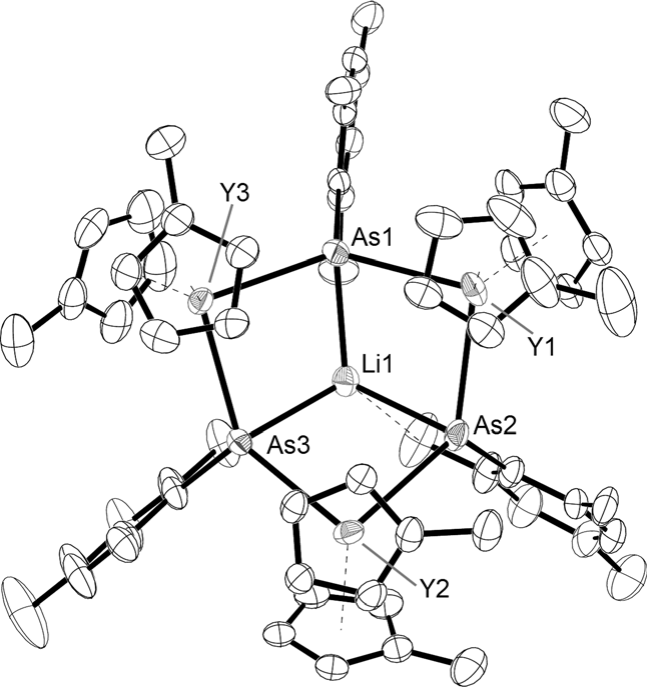
Molecular structure of 3, with ellipsoids set at 50 % probability.^27^ Unlabeled atoms are carbon; hydrogen atoms are not shown.

The ^1^H NMR spectrum of [Li(thf)_4_]_2_[**3**]⋅thf, recorded 30 min after sample preparation in [D_8_]thf at 298 K (Supporting Information, Figure S6), features two resonances at δ(^1^H)=6.71 and 6.80 ppm, both of which integrate to three protons and correspond to two types of mesityl *meta* CH environments. The Cp′ CH protons occur as four resonances at δ(^1^H)=6.44, 6.15, 5.07, and 4.85 ppm, each of which integrates to six protons. Distinct singlets for the *ortho*, *para*, and Cp′ CH_3_ environments were observed in the region δ(^1^H)=1.54–2.62 ppm. The appearance of the ^1^H NMR spectrum of [Li(thf)_4_]_2_[**3**]⋅thf is therefore consistent with the arsinidene complex possessing a *C*_3_ symmetry axis coincident with Li1 and approximately perpendicular to the Y_3_ plane. The ^7^Li NMR spectrum of [Li(thf)_4_]_2_[**3**]⋅thf features two resonances, with a sharp peak at δ(^7^ Li)=−1.64 ppm corresponding to the [Li(thf)_4_]^+^ cations and a broader peak at δ(^1^H)=4.41 ppm corresponding to the {LiAs_3_} environment in [Li(thf)_4_]_2_[**3**]⋅thf (Supporting Information, Figure S8). An additional feature of the ^1^H NMR spectrum of [Li(thf)_4_]_2_[**3**]⋅thf is that, over time, additional resonances which were observed as minor components after 30 min grow in intensity (Supporting Information, Figure S7). After a period of only two hours, the additional resonances account for a significant component of the NMR spectrum. It was not possible to identify the decomposition products; however, this unexpected feature suggests that the arsinidene ligands in [Li(thf)_4_]_2_[**3**]⋅thf react with the thf solvent.

To the best of our knowledge, complex **3** is the first rare-earth metal complex of an arsinidene ligand, which is surprising given that arsinidene ligands are well-known in transition-metal chemistry.^24^ Several alkali metal complexes of arsinidene ligands have also been structurally characterized.^25^ Complex **3** is related to the rare-earth metal phosphinidene complexes, particularly the heterobimetallic lithium–scandium complex [(PNP)Sc(μ-dmp)(μ-Br)Li] (PNP=N(2-*i*Pr_2_PC_6_H_3_-4-Me)_2_, 2,6-Mes_2_C_6_H_3_, dme=dimethoxyethane).^10^ Notably, the arsinidene ligands in **3** adopt a μ-bridging coordination mode, which is an obvious parallel with rare-earth metal phosphinidene complexes.

The variation in the character of the yttrium–arsenic bonding in complexes **1**, **2**, and **3** was investigated using density functional theory: the calculations were simplified by replacing the *para* and Cp′ methyl groups with hydrogen atoms. Geometry optimizations employing two exchange correlation functionals were carried out in the gas-phase and using a continuum dielectric, and the results of the calculations with the hybrid PBE0 functional, including dielectric effects, are described.

Comparing the calculated and experimental Y–As bond lengths, we find good agreement for **1** (3.113 Å vs. 3.095 Å) but also that the calculations slightly overestimate the average distance for **2** (3.061 Å vs. 2.998 Å) and for **3** (2.912 Å vs. 2.872 Å). The discrepancies are most likely due to the inability of the simulations to fully account for solid-state intermolecular interactions, and also the effects of the counter cations on **3**. Despite this, the overall trend in the decrease of the Y–As bond length is reproduced. The atomic charges were calculated by natural bond orbital (NBO) and quantum theory of atoms in molecules (QTAIM) analyses (Table 1). Both types of analysis show an increasing negative charge on the arsenic donor atom on moving from **1** to **2** to **3**. The QTAIM-derived localization index, *λ*, which provides a measure of the number of electrons localized on a given atom, increases by 0.94 from **1** to **2**, and again by 0.90 from **2** to **3**. The analogous parameters for yttrium are essentially constant across the three complexes, which indicates that the observed decrease in Y–As bond lengths is due to stronger ionic interactions. However, the fact that the calculations produce Δ*λ*<1 implies a small-but-increasing non-ionic contribution as the negative charge on arsenic increases.

**Table 1:** Experimental and calculated Y–As bond lengths, atomic charges (*q*) for 1–3, and QTAIM-derived topological parameters at the bond critical points.

	1	2	3
Y–As [Å]^[a]^	3.0945(6)	2.998^[c]^	2.8722^[c]^
Y–As [Å]^[b]^	3.113	3.061^[c]^	2.912^[c]^
*q*_NBO_ (Y/As)	+1.09, +0.42	+1.26, −0.10	+1.13, −0.53
*q*_QTAIM_ (Y/As)	+1.89, +0.86	+1.85, +0.03	+1.82, −0.81
*λ*_QTAIM_	35.86, 30.36	35.89, 31.30	35.89, 32.20
*ρ*	0.024	0.030	0.038
*H*	−0.00577	−0.0338	−0.0592
*δ*(Y,As)	0.200	0.302	0.438

[a] Experimental. [b] PBE0+COSMO. [c] Average length. *λ*_QTAIM_=localization index, *ρ*=electron density (e bohr^−3^), *H*=energy density (a.u.), *δ*=delocalization index.

The Y–As bonding was investigated further by a topological analysis of the electron density. The parameter *ρ*, which describes the electron density at the QTAIM-derived bond critical point (BCP), increases from **1** to **2** to **3**, as expected based on the decreasing Y–As bond lengths, and which is consistent with the change in *λ*. The *ρ* values indicate an enhancement in the non-ionic contribution to the Y–As bonding in the arsinidene complex **3**; however, the values are still markedly less than expected for a typical covalent bond (*ρ*>0.2). The values of the energy density (*H*) at the BCP, and the values of the delocalization indices (*δ*), which provides a measure of the number of electrons shared between yttrium and arsenic, are indicative of considerable ionic bonding character in **1**, **2**, and **3**. However, the general increase across the series also implies an increasing degree of non-ionic character in **3**.

In summary, the synthesis and structure of yttrium complexes with arsine, arsenide, and arsinidene ligands have been described. The synthetic strategy involved initial assembly of an yttrium–arsenic bond, followed by stepwise deprotonation of the {YAsH_2_R} unit. The resulting yttrium–lithium complex [{Cp′_2_Y(μ-AsMes)}_3_Li]^2−^ (**3**) is the first rare-earth metal complex of an arsinidene ligand. As with closely related rare-earth metal phosphinidene complexes, the arsinidene ligands in **3** adopt a μ-bridging coordination mode; stabilization of a terminally bonded [RAs]^2−^ ligand will require greater steric bulk than is provided by the substituents used in the current study. Our computational analysis of the Y–As bonding confirms the expected ionic character, but we also find a small and potentially significant change in non-ionic contributions across the arsine, arsenide, and arsinidene series. The 4f electronic structure of lanthanide(III) cations will be sensitive to such ligand field variations at low temperatures, and thus our study introduces new possibilities for the design of single-molecule magnets.^26^

## Supporting Information

As a service to our authors and readers, this journal provides supporting information supplied by the authors. Such materials are peer reviewed and may be re-organized for online delivery, but are not copy-edited or typeset. Technical support issues arising from supporting information (other than missing files) should be addressed to the authors.

**miscellaneous_information**
