# Supplementary material for: Yttrium Complexes of Arsine, Arsenide, and Arsinidene Ligands
Source: Angew Chem Int Ed Engl. 2015 Feb 5;54(14):4255–8. doi: 10.1002/anie.201500173 (PMC4672686; doi:10.1002/anie.201500173)
Supplement: Supplementary file 1 [file anie0054-4255-sd1.pdf]

Angewandte Chemie
Eine Zeitschrift der Gesellschaft Deutscher Chemiker

Supporting Information

Yttrium Complexes of Arsine, Arsenide, and Arsinidene Ligands**

*Thomas Pugh, Andrew Kerridge, and Richard A. Layfield**

anie_201500173_sm_miscellaneous_information.pdf

General experimental considerations. All syntheses were carried out using standard Schlenk and glove-box techniques, using an inert atmosphere of nitrogen or argon. Toluene and thf were dried by refluxing over molten potassium, followed by distillation and storage over a potassium mirror and activated 4 Å molecular sieves, respectively; all solvents were freeze-thaw degassed prior to use. Benzene-D_6 was distilled off molten potassium, vacuum transferred to an ampoule and freeze-thaw degassed prior to use. X-ray diffraction data for complexes **1** and **2**·toluene were collected on an Oxford Instruments XCalibur2 diffractometer or an Agilent SuperNova, using MoKα radiation, the data for $[Li(thf)_4]_2$[**3**]·thf was collected on a Bruker APEX-II diffractometer, using CuKα radiation. NMR spectra were acquired on a Bruker Avance-III 400 MHz spectrometer at 400.13 MHz (^{1}H) and 155.51 MHz (^{7}Li). ^{1}H chemical shifts were referenced internally to residual solvent resonances (relative to $SiMe_4$) and ^{7}Li chemical shifts were referenced to an external standard of aqueous LiCl. FTIR spectra were recorded as Nujol mulls in KBr discs on a Perkin Elmer Spectrum RX1 spectrometer. Elemental analyses were carried out at London Metropolitan University, U.K.

Synthesis of Cp′$_3$Y. Cp′$_3$Y was synthesised using a modified literature procedure.[1] A solution of NaCp′ (4.08 g, 40 mmol) in thf (100 ml) was added to suspension a of $[YI_3(thf)_{3.5}]$[2] (13.3 mmol) in thf (50 ml), and the reaction was warmed to room temperature overnight, followed by refluxing for three hours. The thf was removed *in vacuo* and the solid residue was sublimed onto a water-cooled finger (180°C, 2 × 10^{-2} mbar); to remove the residual thf, two or three re-sublimations are generally necessary. Cp′$_3$Y was obtained as a pale yellow solid (2.52 g, 77%). Elemental analysis for $C_{18}H_{21}Y$: calculated C 66.26 H 6.49; found C 66.13, H 6.39. Melting point 160-163°C. ^{1}H NMR (benzene-D_6, 298.15 K, δ/ppm): 5.94, 5.84 (m, 12H, Cp′ CH); 1.95 (s, 9H, CH_3).

Synthesis of MesAsH$_2$. Mesitylarsine was synthesized according to a modified version of a literature procedure,[3] in two steps:

Step 1: synthesis of $(Me_2N)_2AsCl$. A solution of $AsCl_3$ (5.0 ml, 59 mmol) in ether (50 ml) was cooled to –78°C and a suspension of $LiNMe_2$ (6.04 g, 112 mmol) in ether (100 ml) was added dropwise, with vigorous stirring. The reaction was slowly warmed to room temperature, stirred for 18 hours, and filtered. The solid residue was washed with ether (20 ml), and the solvent was evaporated from the combined washings to leave a colourless oil. The crude product was distilled (140°C, 0.1 mbar), which gave $(Me_2N)_2AsCl$ as a colourless oil (9.60 g, 82%). Purity was established by ^{1}H NMR spectroscopy (benzene-D_6): $\delta(^1H)$ = 2.47 (s, 12 H, NMe_2).

Step 2. A solution of MesMgBr (1.0 M in thf, 25.3 ml, 25 mmol) was added to thf (40 ml) solution of $(Me_2N)_2AsCl$ (5.00 g, 25 mmol) at –78°C. After slowly warming the reaction to room temperature and stirring for 18 hours, the solvent was removed and replaced with hexane (50 ml). After filtration and washing of the residue with hexane (2 × 20 ml), the solvent was removed in vacuo, leaving a pale yellow oil. The yellow oil was dissolved in ether (30 ml), cooled to –78°C and a solution of HCl in ether (2.0 M, 50.6 ml, 101 mmol) was added dropwise. The reaction was slowly warmed to room temperature, stirred overnight, filtered, and the solid residue washed with ether (20 ml). The ether was removed *in vacuo*, which gave a colourless solid. The solid was re-dissolved in ether (50 ml), cooled to –78°C and $LiAlH_4$ (1.92 g, 51 mmol) in ether (100 ml) was added dropwise. After slowly warming the reaction to room temperature and stirring overnight, the solvent was removed and replaced with pentane (50 ml). After filtration and washing of the residue with pentane (20 ml), the solvent was removed in vacuo, leaving an oily residue. The oil was distilled (70°C, 0.1 mbar), giving MesAsH$_2$ as a colourless oil (2.11 g, 42% based on arsenic). The ^{1}H NMR chemical shifts of the product are identical to those previously reported.[3]

Synthesis of 1. Mesitylarsine (0.5 M in toluene, 2.5 ml, 1.25 mmol) was added to a solution of Cp′$_3$Y (0.40 g, 1.23 mmol) in toluene (5 ml) and the mixture was stirred for one hour. The solution was concentrated and stored overnight at –30°C, which resulted in the formation of **1** as colourless crystals (0.56 g, 88%). Elemental analysis for $C_{27}H_{34}AsY$: calculated C 62.08 H 6.56; found C 61.89, H 6.47. Melting point 69-70°C. ^{1}H NMR (toluene-D_8, 298.15 K, δ/ppm): 6.67 (s, 2H, mesityl CH), 5.86, 5.77 (s, 12H, C_5H_4); 3.12 (s, 2H, AsH); 2.18 (s, 6H, *ortho* CH_3); 2.08 (s, 3H, mesityl *para* CH_3); 1.95 (s, 9H, Cp′ CH_3).

Synthesis of 2·toluene. A freshly prepared solution of **1** (0.50 g, 1.0 mmol) in toluene (20 ml) was cooled to –78°C and ^{n}BuLi (1.6 M in hexanes, 0.6 ml, 1.0 mmol) was added dropwise. The reaction mixture was slowly warmed to room temperature overnight. The resulting cloudy mixture was briefly heated to dissolve a

cloudy precipitate, and then the pale yellow solution was filtered to remove a gelatinous precipitate of Cp′Li. The yellow solution was concentrated and stored overnight at –30°C, which resulted in the formation of **2**·toluene as colourless crystals (0.27 g, 59% based on yttrium). Elemental analysis for $C_{70}H_{86}As_3Y_3$: calculated C 59.25 H 6.11; found C 59.12, H 6.07. Melting point: decomposes to a black solid at 224°C. ^{1}H NMR (toluene-D$_8$, 298.15 K, δ/ppm): 6.94-6.78 (singlets, 6H, mesityl CH); 6.51-5.84 (multiplets, 24H, Cp' CH); 2.66, 2.65, 2.48, 2.39 (s, 18H, mesityl *ortho* CH_3); 2.60, 2.51 (s, 1H and 2H, AsH); 2.15 (s, 9H, mesityl *para* CH_3); 2.12 (s, 3H, toluene of solvation); 2.04-1.72 (Cp' CH_3).

Synthesis of [Li(thf)$_4$]$_2$[3]·thf. A solution of **2**·toluene (0.30 g, 0.21 mmol) in thf (15 ml) was cooled to –10°C and ^{n}BuLi (1.6 M in hexanes, 0.40 ml, 0.64 mmol) was added dropwise. The reaction mixture was slowly warmed to room temperature and stirred for one hour, which resulted in the formation of a bright orange solution. Concentration of the solution to a volume of ca. 4 ml, followed by storage at 4°C overnight, resulted in the formation of large orange crystals of [Li(thf)$_4$]$_2$[**3**]·thf (0.26 g, 62% based on yttrium). A second crop of crystals was obtained by layering the nascent solution with hexane, resulting in a total yield of 0.31 g, 73%. Elemental analysis for $C_{99}H_{147}As_3Li_3O_9Y_3$: calculated C 59.64, H 7.43; found C 59.41, H 7.34. Melting point 138-139°C, with slow decomposition to a black solid. ^{1}H NMR (thf-D$_8$, 298.15 K, δ/ppm): 6.80, 6.71 (2 × s, 2 × 3H, mesityl CH); 6.44, 6.15, 5.07, 4.86 (4 × s, 4 × 6H, Cp' CH); 3.62 (thf CH_2O); 2.62, 2.56 (2 × s, 2 × 9H, mesityl *ortho* CH_3); 2.35 (s, 9H, mesityl *para* CH_3); 2.15, 1.54 (2 × s, 2 × 9H, Cp' CH_3); 1.78 (thf $C\underline{H}_2CH_2O$). ^{7}Li NMR (thf-D$_8$): 4.41 (As_3Li), –1.64 (Li[thf]$_4$).

Table S1. Crystal data and structure refinement for **1**, **2**·toluene and $[Li(thf)_4]_2$[**3**]·thf

	**1**	**2**·toluene	$[Li(thf)_4]_2$[**3**]·thf[a]
CCDC ref. code	953898	953899	953900
Formula	$C_{27}H_{34}AsY$	$C_{70}H_{83}As_3Y_3$	$C_{99}H_{147}As_3Li_3O_9Y_3$
FW	522.37	1415.85	1993.47
T / K	150	150	100
Crystal system	monoclinic	monoclinic	monoclinic
Space group	$P2_1/n$	Cc	$P2_1/c$
a/Å	8.1991(3)	14.9207(9)	22.3495(2)
b/Å	24.7652(10)	18.5911(11)	15.73082(16)
c/Å	11.6296(5)	23.7077(15)	28.0040(3)
α/°	90	90	90
β/°	94.396(4)	103.477(6)	95.6020(9)
γ/°	90	90	90
V/Å^3	2354.48(17)	6395.2(7)	9798.50(17)
Z	4	4	4
Crystal size/mm^3	0.1 × 0.05 × 0.02	0.3 × 0.3 × 0.3	0.2 × 0.1 × 0.05
θ range/°	3.7-26.4	3.8 to 25.4	2.0 to 68.3
Reflections collected	15831	21361	53993
Independent reflections, R(int)	4802 [R_{int} = 0.0333, R_{sigma} = 0.0348]	10335 [R_{int} = 0.0433, R_{sigma} = 0.0607]	17686 [R_{int} = 0.0246, R_{sigma} = 0.0183]
Completeness/%	99.7	1.76/0.88	98.7
Data/restraints/parameters	4802/122/287	10335/174/701	17686/1632/1290
Goodness-of-fit on F^2	1.045	1.026	1.170
Final R indices [I>2σ(I)]	R_1 = 0.0301<br>wR_2 = 0.0641	R_1 = 0.0521<br>wR_2 = 0.1069	R_1 = 0.0532<br>wR_2 = 0.1229
R indices (all data)	R_1 = 0.0437<br>wR_2 = 0.0691	R_1 = 0.0648<br>wR_2 = 0.1141	R_1 = 0.0543<br>wR_2 = 0.1234

Structures were solved using SHELXS, and SHELXL was used for the refinement within Olex2.[4]

[a] The high number of restraints/constraints used in this model reflects the extensive disorder present in both the thf and the Cp′ ligands.

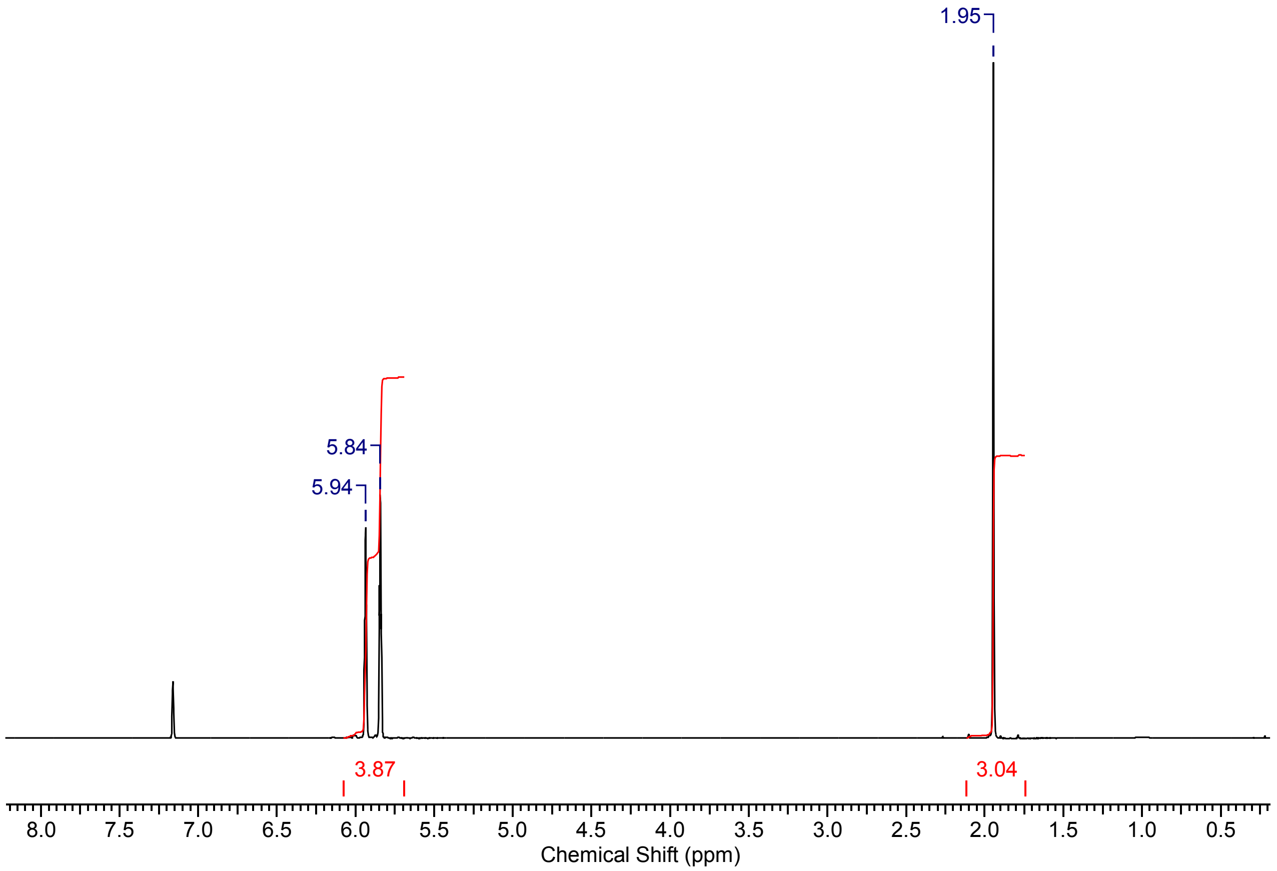

Figure S1. ^{1}H NMR spectrum of Cp'_3Y in benzene-D_6 at 298 K.

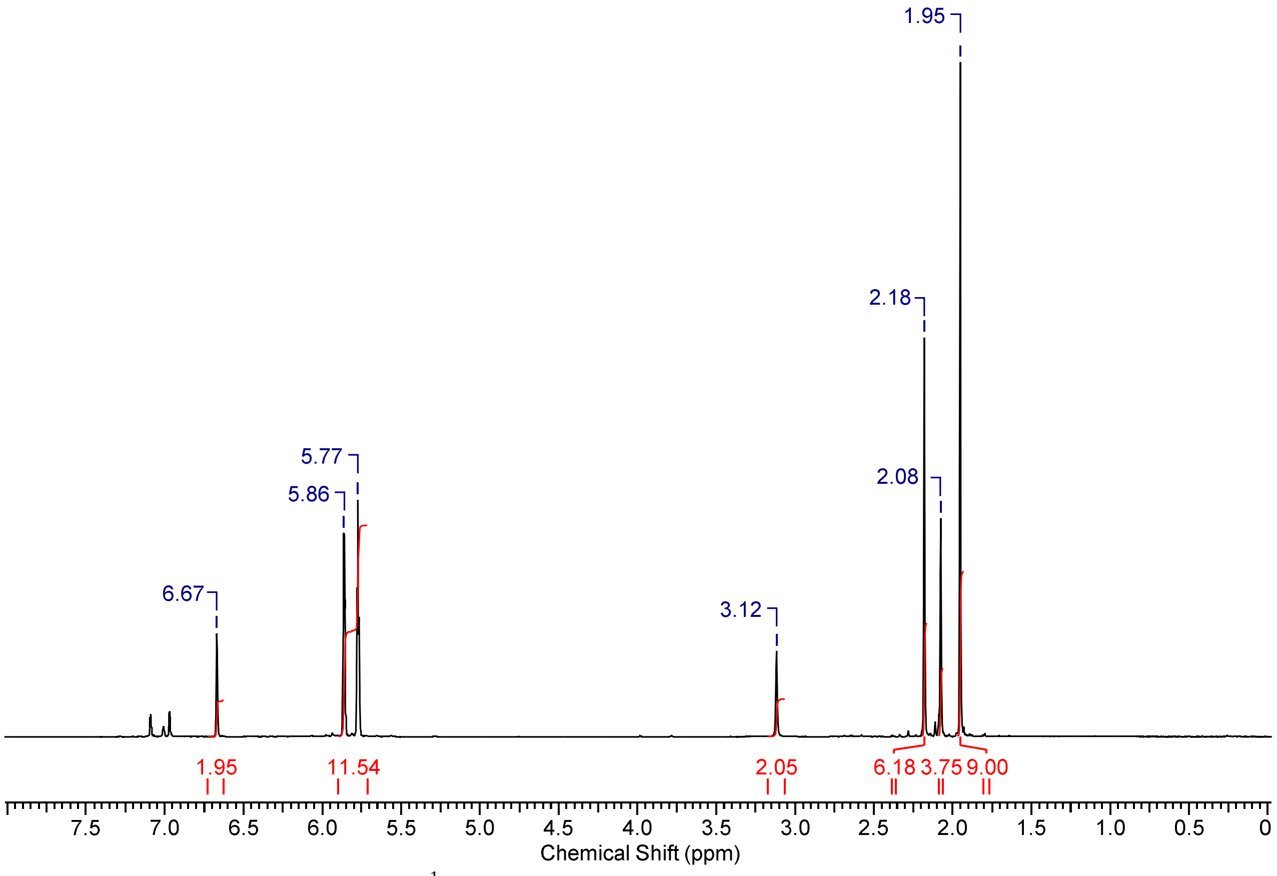

Figure S2. ^{1}H NMR spectrum of **1** in toluene-D_8 at 298 K.

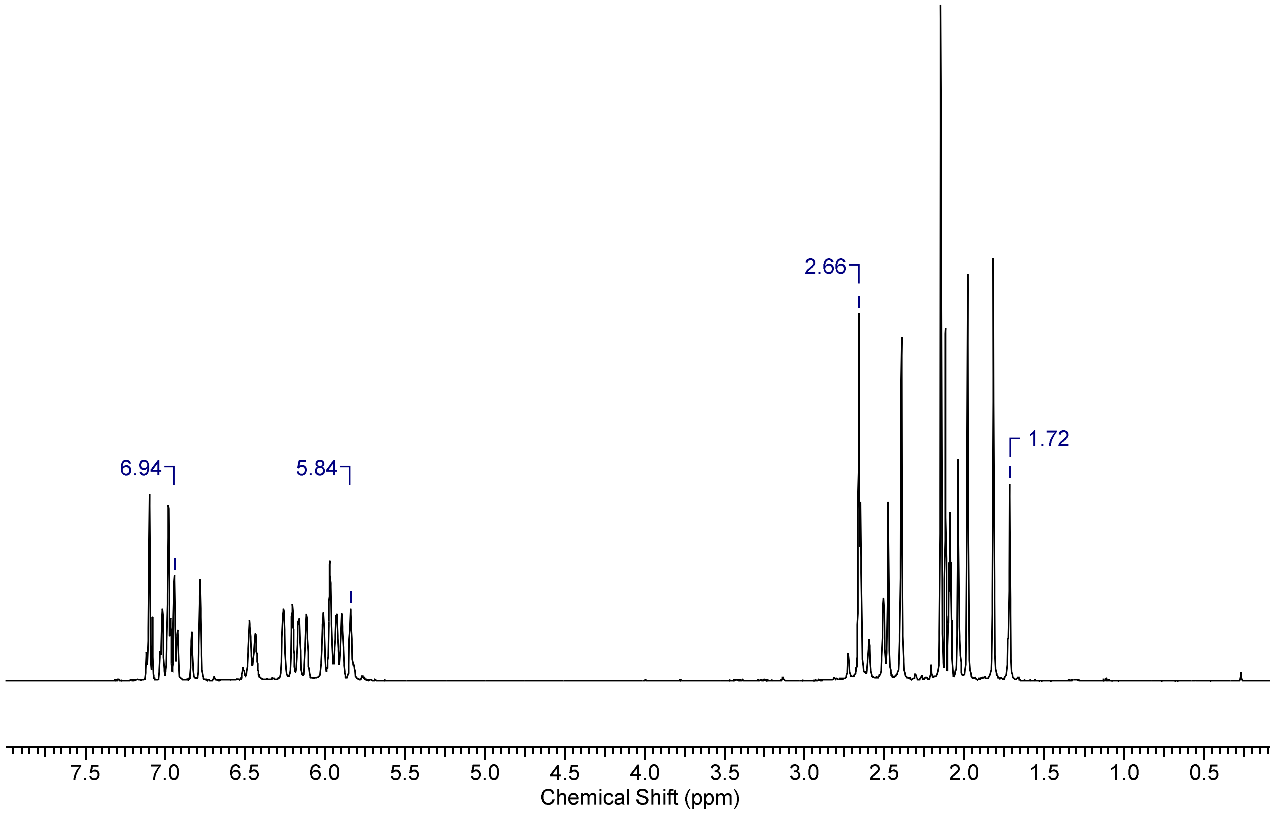

Figure S3. ^{1}H NMR spectrum of **2**·toluene in toluene-D_6 at 298 K.

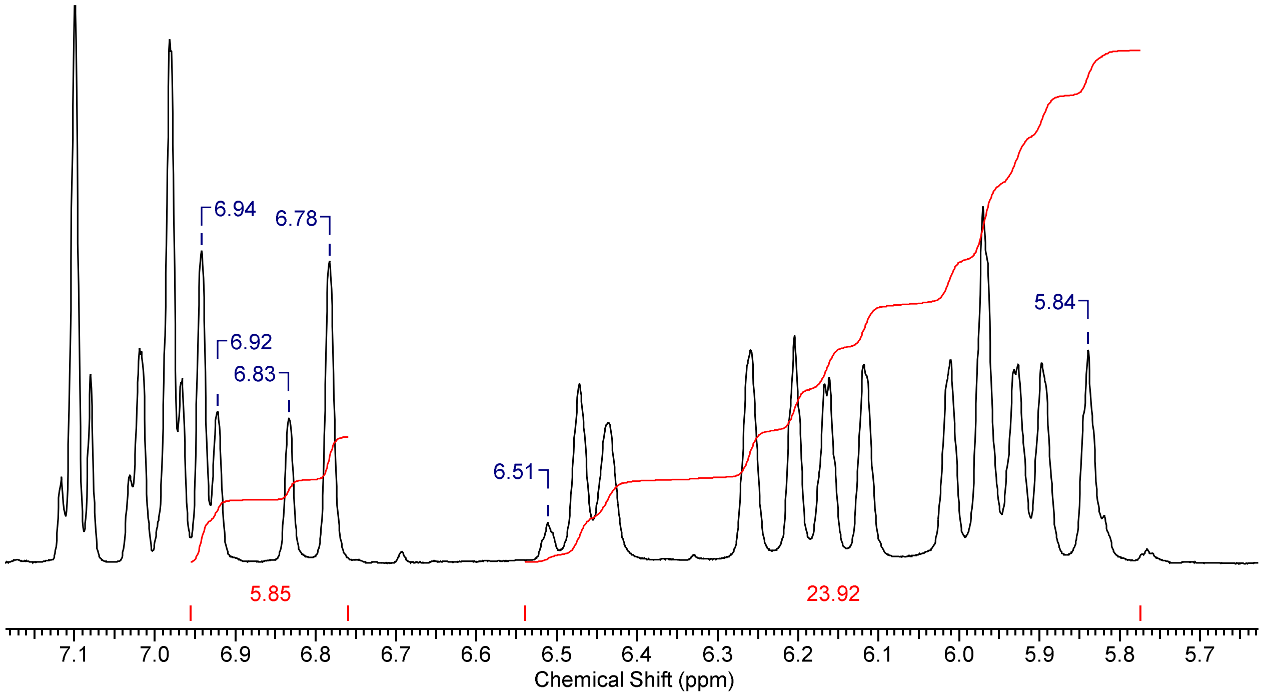

Figure S4. Expanded section of the ^{1}H NMR spectrum of **2**·toluene in toluene-D_8 at 298 K.

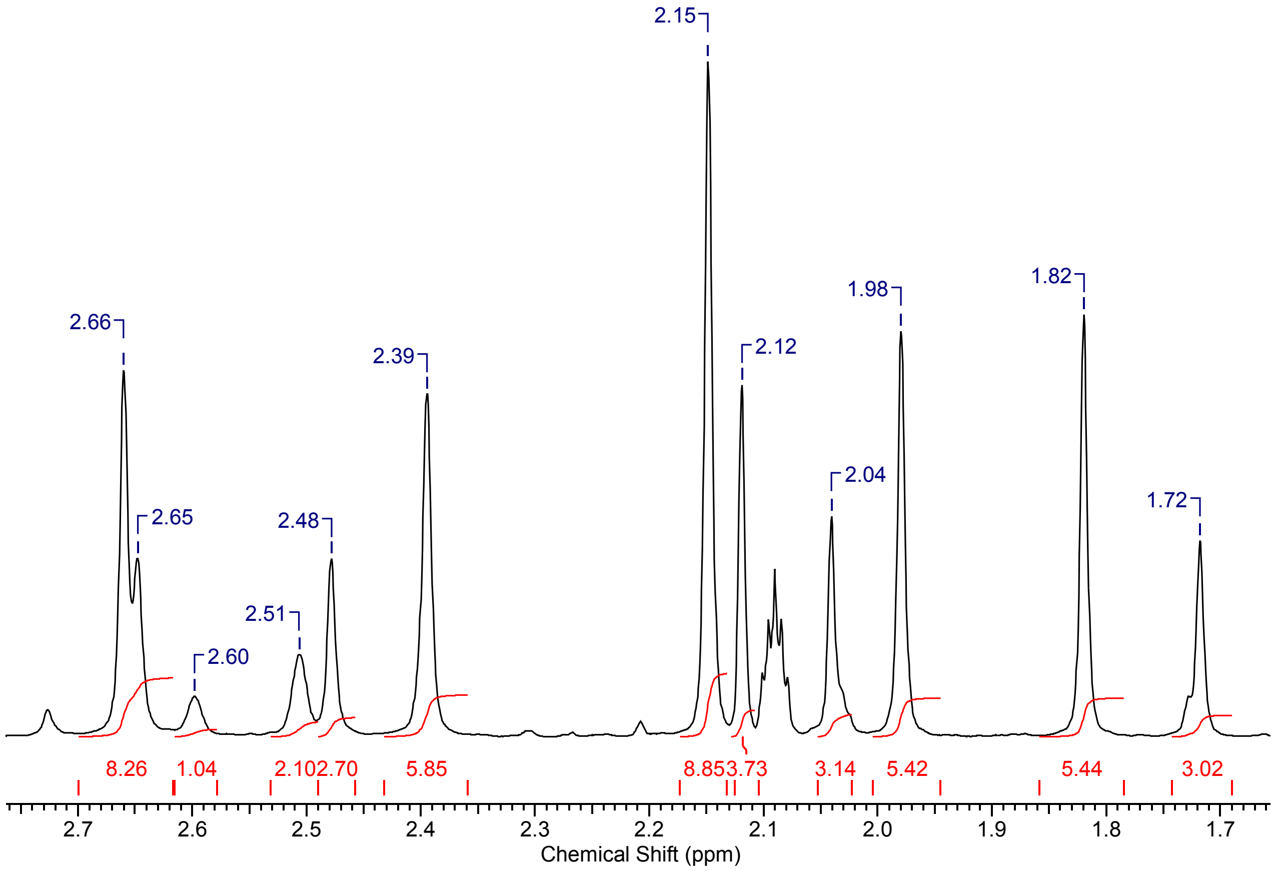

Figure S5. Expanded section of the ^{1}H NMR spectrum of **2**·toluene in toluene-D_8 at 298 K.

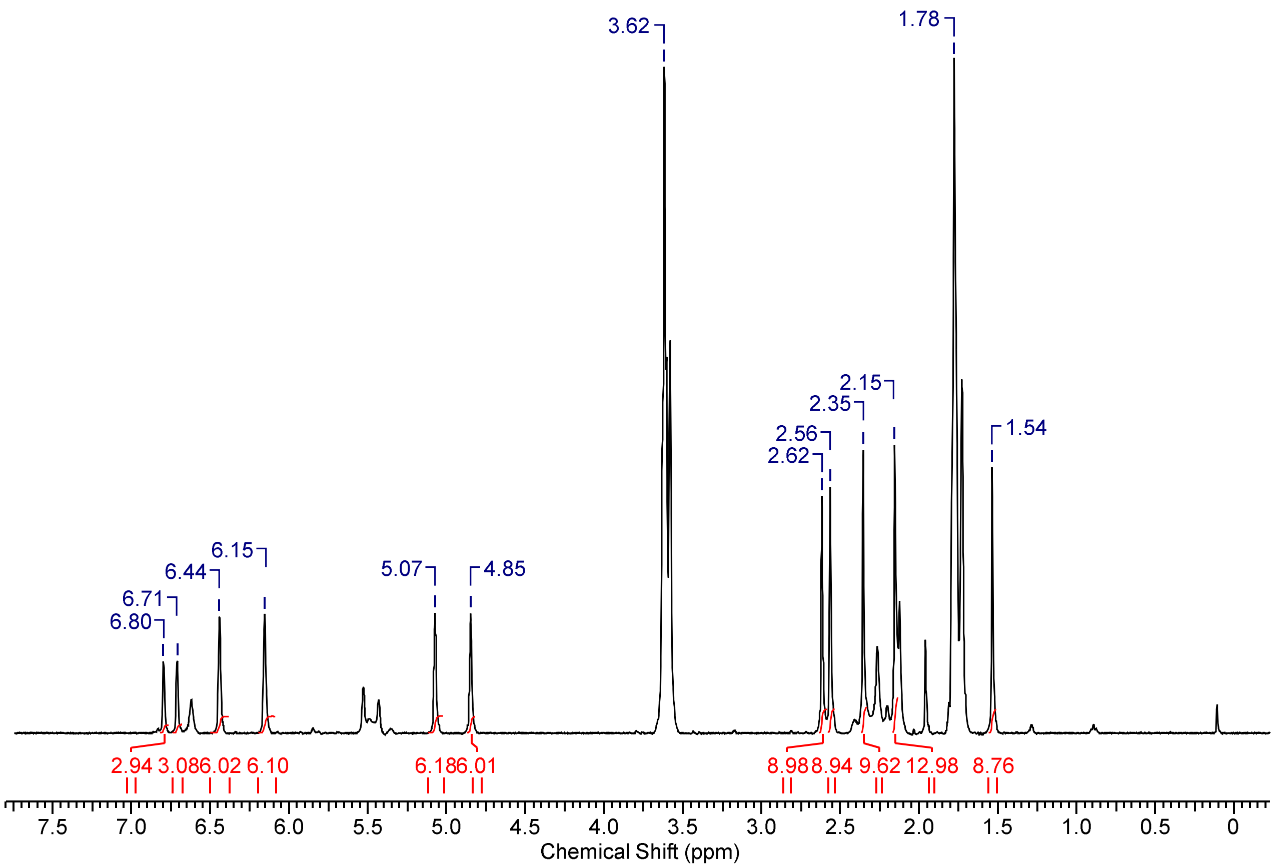

Figure S6. ^{1}H NMR spectrum of $[Li(thf)_4]_2$[**3**]·thf in thf-D_8 at 298 K.

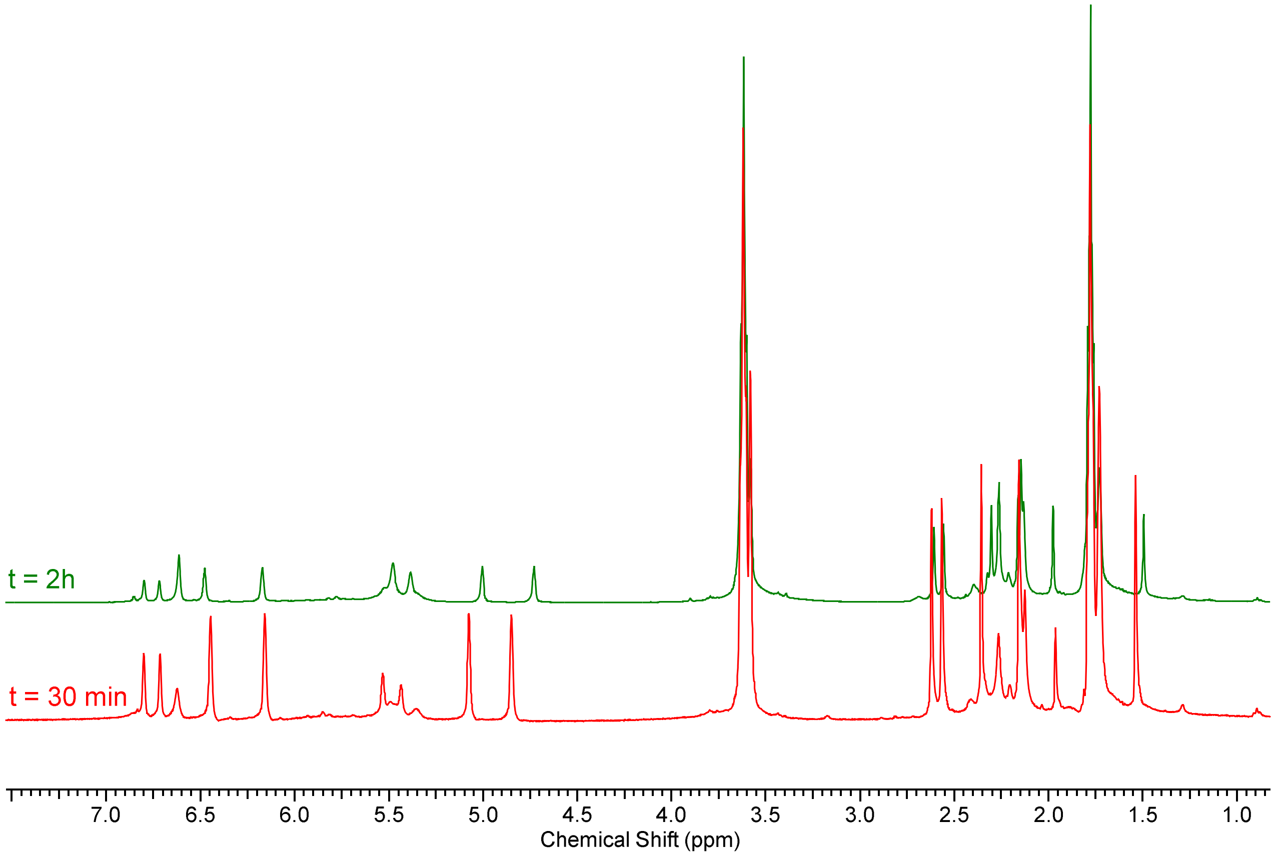

Figure S7. ^{1}H NMR spectrum of $[Li(thf)_4]_2$[**3**]·thf in thf-D_8 at 298 K recorded 30 minutes after sample preparation, and after 2 hours.

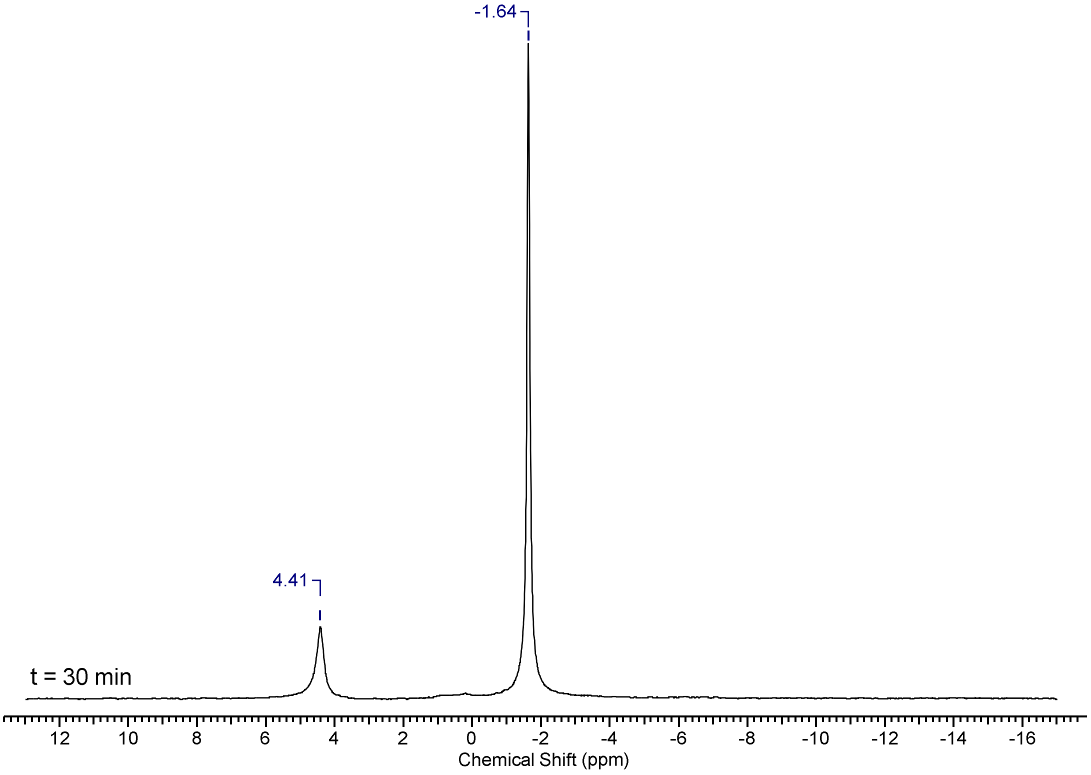

Figure S8. ^{7}Li NMR spectrum of $[Li(thf)_4]_2$[**3**]·thf in thf-D_8 at 298 K recorded 30 minutes after sample preparation.

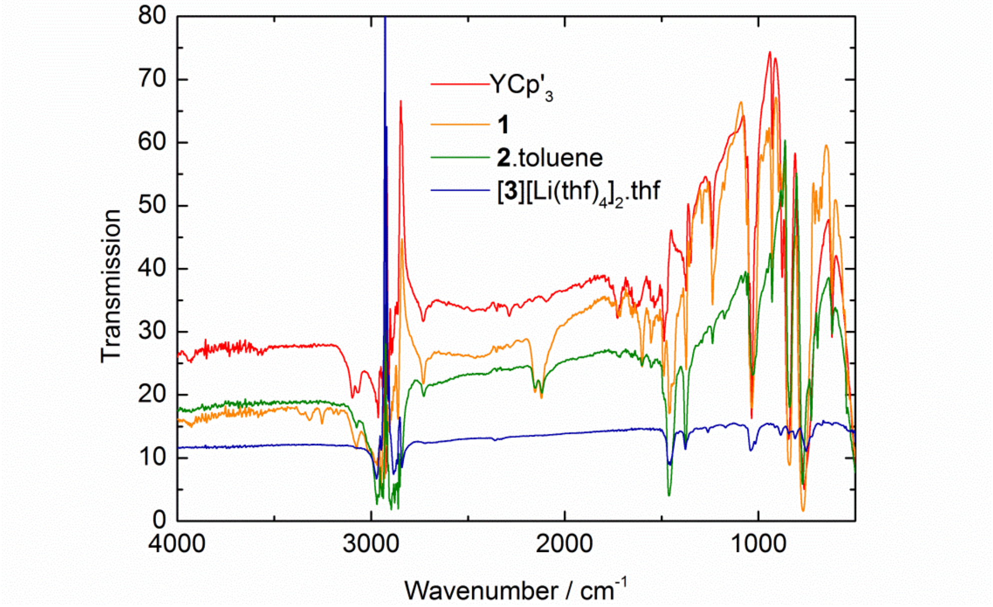

Figure S9. Infrared spectrum (Nujol mulls) of Cp'_3Y, **1**, **2**·toluene and $[Li(thf)_4]_2$[**3**]·thf.

Computational details

All calculations were performed at the density functional theoretical (DFT) level using version 6.6 of the TURBOMOLE quantum chemistry software package[5]. Atomic coordinates from crystallography were used as the starting point for geometry optimisations of **1**, **2** and **3**. To simplify the calculations, the mesityl *para* and Cp′ CH_3 groups were replaced by hydrogen atoms, ensuring that any important steric/electronic effects would be preserved. To establish any functional dependence, two exchange correlation functionals were used: the pure PBE functional[6], employing the generalized gradient approximation (GGA) and the hybrid-GGA PBE0 functional[7], which incorporates a perturbatively derived 25% contribution of exact exchange. In all calculations, basis sets of polarised triple-ζ quality were used. For geometry optimisations, the Ahlrichs def2-TZVP basis sets[8] were employed, incorporating an effective core potential replacing 28 core electrons of the yttrium ion. For subsequent natural bond order and topological analysis, all-electron single point energy calculations were performed at the optimised geometries. These calculations replaced the yttrium def2-TZVP basis with the all-electron TZVPalls[29] set. Optimisations were performed in the gas phase and in the presence of a continuum dielectric, the latter chosen so as to stabilise the excess negative charge in complex **3**. The continuum dielectric also served to give a simple approximation to the effects of counterions. Analytical vibrational frequency analysis was performed on all gas-phase structures. No imaginary frequency modes were found, indicating that the optimised structures are energetic minima. Equivalent numerical analysis in the presence of the continuum solvent proved computationally intractable, however the strong similarities in all computed properties between the solvated and gas phase complexes, along with the fact that low-frequency vibrational modes in the gas phase were primarily associated with motion of the Cp′ and mesityl ligands, lead us to infer that the latter are also energetic minima. The resulting electron densities were studied using natural bond order (NBO) analysis[10] and the Quantum Theory of Atoms in Molecules[11] (QTAIM) using version 6.0 of the NBO and version 14.11.23 of the AIMAll codes, respectively.[12,13]

Table S2. Calculated and experimental Y–As bond lengths [Å] in complexes **1-3**. GP = gas-phase optimisations, COSMO = optimisations in the presence of a continuum dielectric.

	**1**	**2**[a]	**3**[a]
PBE GP	3.127	3.081	2.941
PBE COSMO	3.128	3.077	2.927
PBE0 GP	3.126	3.065	2.929
PBE0 COSMO	3.113	3.061	2.912
Experiment	3.095	2.998	2.872

[a] average of six Y–As bonds

Table S3. Differences in calculated and experimental Y–As bond lengths [Å] in complexes **1-3**.

	**1-2**	**1-3**	**2-3**
PBE GP	0.046	0.186	0.140
PBE COSMO	0.051	0.201	0.150
PBE0 GP	0.061	0.197	0.136
PBE0 COSMO	0.052	0.201	0.149
Experiment	0.097	0.223	0.126

Table S4. QTAIM and NBO atomic charges, along with QTAIM delocalisation indices. All values correspond to PBE0-COSMO calculated densities.

	**1**		**2**		**3**	
	Y	As	Y	As	Y	As
q_{NBO}	+1.09	+0.42	+1.26	-0.10	+1.09	-0.58
q_{QTAIM}	+1.89	+0.86	+1.85	+0.03	+1.82	-0.81
λ_{QTAIM}	35.86	30.36	35.89	31.30	35.89	32.20

Table S5. Natural electron configurations for complexes **1-3**.

		**1**	**2**	**3**
Y	5s	0.18	0.24	0.24
	4d	1.70	1.48	1.66
	5d	0.02	0.02	0.02
As	4s	1.48	1.55	1.58
	4p	3.08	3.53	3.99
	4d	0.01	0.01	0.01

Table S6. QTAIM-derived topological parameters obtained at the Y–As bond critical points. ρ = electron density (e/bohr^3), $\nabla^2\rho$ = Laplacian of electron density (e/bohr^5), H = energy density (a.u.).

	**1**	**2**	**3**
ρ	0.024	0.030	0.038
$\nabla^2\rho$	0.054	0.044	0.055
10^3H	–0.577	–3.38	–5.92
δ(Y, As)	0.200	0.302	0.438

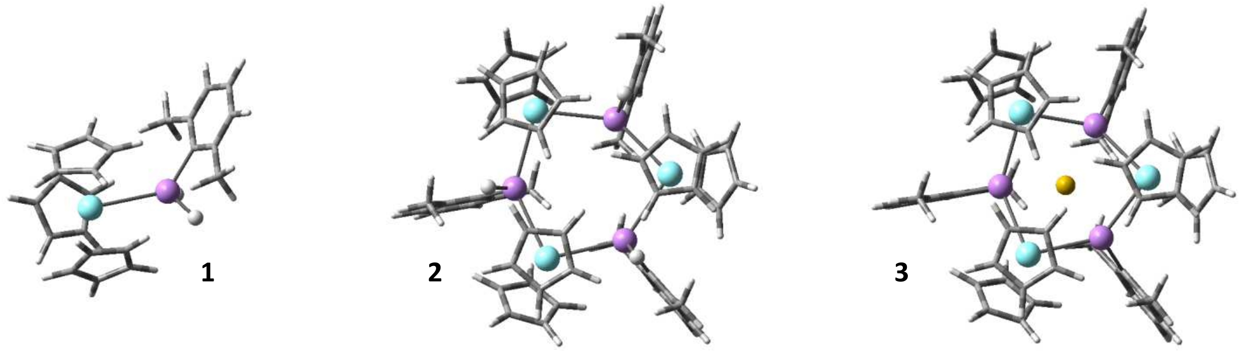

Figure S10. PBE0-COSMO optimised molecular structures of **1-3**. Y = cyan, As = purple, Li = orange.

PBE0-COSMO optimised coordinates

1

Y	0.6210284	-0.7762336	1.4914594
C	-1.0558698	-0.5855773	3.5906860
H	-1.7419618	-1.4083257	3.7219468
C	-1.2900910	0.5788029	2.8436457
H	-2.1983641	0.8204438	2.3098257
C	-0.1441314	1.4052011	2.9606921
H	-0.0293936	2.3976885	2.5481449
C	0.7849321	0.7472101	3.7774694
H	1.7560956	1.1234198	4.0598416
C	0.2390173	-0.4987369	4.1459292
C	0.1896879	-3.1836842	0.3860996
H	1.0164114	-3.8767276	0.4269553
C	-0.0811414	-2.2765053	-0.6505062
H	0.4906054	-2.1549010	-1.5592260
C	-1.2709444	-1.5842239	-0.3206625
H	-1.7712678	-0.8604447	-0.9482681
C	-1.7269941	-2.0653930	0.9150177
H	-2.6166209	-1.7385324	1.4316394
C	-0.8134578	-3.0384773	1.3693678
C	3.1227172	-0.8791109	2.4626806
H	3.2088442	-0.8722819	3.5388856
C	3.1263328	0.2457874	1.6222297
C	3.0210452	-0.2127534	0.2879965
H	3.0569366	0.4065398	-0.5967646
C	2.9555151	-1.6147238	0.3100838
H	2.8762640	-2.2599179	-0.5515791
C	2.9956525	-2.0303712	1.6553422
As	0.1432365	1.6058111	-0.4755007
C	-0.8052758	1.5134333	-2.1779945
C	-2.1837209	1.7622766	-2.2188195
C	-2.8444980	1.6451571	-3.4381735
H	-3.9115735	1.8358941	-3.4786797
C	-2.1641471	1.2825494	-4.5867579
C	-0.8071336	1.0199271	-4.5282165
H	-0.2741547	0.7198299	-5.4240396
C	-0.1087974	1.1242875	-3.3292782
C	-2.9663702	2.1444875	-0.9981694
H	-2.7897141	1.4546758	-0.1673336
H	-4.0356578	2.1401342	-1.2102221
H	-2.7009056	3.1462578	-0.6469483
C	1.3581790	0.8185364	-3.3035074
H	1.9534928	1.7199216	-3.1271764
H	1.6811380	0.3893228	-4.2521681
H	1.6091624	0.1049457	-2.5127767
H	1.3631112	2.3760920	-0.9361129
H	-0.5555388	2.8188304	0.0970779
H	-2.6952547	1.1924544	-5.5273745

H	0.7026184	-1.2328528	4.7903931
H	-0.8950110	-3.6142034	2.2809248
H	2.9850445	-3.0520411	2.0083347
H	3.2209227	1.2761010	1.9335867

2

Y	-0.5730646	2.7004550	1.9761880
Y	2.8735436	0.2851279	-1.7838395
Y	-0.1895813	-2.9292795	1.7036719
As	1.0093025	2.4297961	-0.6346646
As	1.3157649	-2.1885627	-0.8623841
As	-1.5022928	-0.2090287	2.2301130
C	1.9334512	2.9172627	2.8395143
H	2.7332115	2.9523503	2.1157964
C	1.4274447	1.7613161	3.4643731
H	1.7678805	0.7489415	3.3038183
C	0.4242280	2.1641400	4.3736448
H	-0.1446397	1.5144304	5.0228515
C	0.3073473	3.5634565	4.3029817
H	-0.3660688	4.1734972	4.8869083
C	1.2329488	4.0314197	3.3448670
C	-3.1514024	3.1816400	1.6647051
H	-3.8391104	2.3514669	1.7413089
C	-2.5443128	3.6619836	0.4874209
H	-2.6902946	3.2845324	-0.5139038
C	-1.7382459	4.7699732	0.8288118
H	-1.1568593	5.3600474	0.1342755
C	-1.8485204	4.9751059	2.2164575
H	-1.3639202	5.7572281	2.7825978
C	-2.7122891	3.9863258	2.7377581
C	4.4553981	-0.6600079	0.1342338
H	4.2129511	-1.6077082	0.5898884
C	4.0463260	0.6090315	0.5867732
H	3.4306954	0.8094859	1.4512770
C	4.6209123	1.5786842	-0.2646524
H	4.5145568	2.6498397	-0.1729251
C	5.3749204	0.9080737	-1.2436968
H	5.9468395	1.3740630	-2.0326959
C	5.2646263	-0.4794357	-1.0057816
C	2.0910857	1.2774478	-4.1034919
H	1.4212674	2.1256752	-4.0973861
C	1.7224451	-0.0818626	-4.1434009
H	0.7184325	-0.4767340	-4.1919594
C	2.9024164	-0.8572437	-4.1611334
H	2.9427784	-1.9365874	-4.2062948
C	3.9999444	0.0228357	-4.1337061
H	5.0419576	-0.2615287	-4.1548690
C	3.5007685	1.3434560	-4.0854274
C	1.1693039	-2.0844885	3.8294429
H	0.8577050	-1.1754532	4.3206618

C	2.1243259	-2.1880357	2.7999104
H	2.6758369	-1.3714957	2.3576296
C	2.2683532	-3.5560043	2.4786167
H	2.9404756	-3.9693206	1.7406775
C	1.3984034	-4.2919509	3.3022293
H	1.2866565	-5.3662875	3.3060246
C	0.7079426	-3.3817016	4.1320988
C	-1.5506702	-4.5158596	0.0887605
H	-1.2594779	-4.5590955	-0.9511572
C	-2.5127153	-3.6565804	0.6554827
H	-3.1139574	-2.9273444	0.1329265
C	-2.6011077	-3.9444367	2.0350799
H	-3.2658063	-3.4585312	2.7356911
C	-1.6949985	-4.9823975	2.3203282
H	-1.5380672	-5.4400211	3.2862308
C	-1.0350025	-5.3290770	1.1205629
C	0.3004148	3.6874573	-1.9611695
C	0.7900519	4.9978591	-2.1104542
C	0.2097972	5.8293143	-3.0661015
H	0.5903989	6.8393824	-3.1778928
C	-0.8343746	5.3991355	-3.8648159
C	-1.3128606	4.1106954	-3.7136949
H	-2.1312409	3.7582244	-4.3330346
C	-0.7572630	3.2482237	-2.7743027
C	1.9106660	5.5554864	-1.2841732
H	1.6825289	5.5233798	-0.2147048
H	2.1025035	6.5942688	-1.5563149
H	2.8386000	4.9945724	-1.4274057
C	-1.2841837	1.8522110	-2.6657059
H	-0.5383347	1.1292726	-3.0073007
H	-2.1826255	1.7187836	-3.2699192
H	-1.5242361	1.5849818	-1.6332105
C	0.8815077	-3.3056341	-2.4141902
C	-0.3231812	-3.0453162	-3.0879603
C	-0.6655620	-3.7949309	-4.2087003
H	-1.5982802	-3.5810494	-4.7204760
C	0.1630036	-4.8011874	-4.6700122
C	1.3478562	-5.0582126	-4.0041486
H	2.0043785	-5.8452247	-4.3607372
C	1.7289208	-4.3271114	-2.8809583
C	-1.2628885	-1.9855787	-2.6057378
H	-1.6993642	-2.2627310	-1.6422273
H	-2.0796197	-1.8285487	-3.3116843
H	-0.7557405	-1.0295438	-2.4519898
C	3.0362487	-4.6714329	-2.2315450
H	3.7138275	-3.8130936	-2.2008668
H	3.5329690	-5.4730365	-2.7798307
H	2.9009261	-5.0056377	-1.1989211
C	-3.4470134	-0.4353809	2.3369312
C	-4.1801037	-0.3449668	1.1421164

C	-5.5594352	-0.5247844	1.1608129
H	-6.1122228	-0.4548173	0.2296110
C	-6.2252588	-0.7868779	2.3439415
C	-5.5027590	-0.8713680	3.5204694
H	-6.0178452	-1.0773074	4.4531690
C	-4.1199373	-0.7023475	3.5432900
C	-3.5044595	-0.0321262	-0.1556783
H	-3.1220861	0.9922961	-0.1566648
H	-4.1927279	-0.1341777	-0.9959636
H	-2.6454148	-0.6834725	-0.3369509
C	-3.4214806	-0.8213101	4.8651797
H	-2.6621281	-1.6087051	4.8548192
H	-4.1381216	-1.0551543	5.6536700
H	-2.9121665	0.1068497	5.1399142
H	2.1446541	3.3004543	-0.1525303
H	-1.2358442	-0.2611427	3.7154766
H	2.5634972	-2.9336602	-0.4525545
H	-7.3001369	-0.9256526	2.3504568
H	-1.2723482	6.0652902	-4.5990582
H	-0.1108068	-5.3810666	-5.5437671
H	1.4025106	5.0643704	3.0753081
H	-3.0142952	3.8840986	3.7707352
H	-0.0165845	-3.6371837	4.8924482
H	-0.2921023	-6.1056470	1.0030472
H	5.7475985	-1.2626233	-1.5729289
H	4.0921316	2.2484054	-4.0743328

3

Y	-1.0390694	-0.9292808	2.9609172
C	-1.1982752	-3.5889745	2.5369804
H	-0.3007155	-4.0447010	2.1478972
C	-2.2431186	-3.0315449	1.7768096
H	-2.3082036	-2.9992985	0.7001438
C	-3.2047550	-2.5087205	2.6687672
H	-4.1146333	-1.9990660	2.3871721
C	-2.7509057	-2.7388186	3.9786871
H	-3.2588887	-2.4461630	4.8870442
C	-1.5025678	-3.3981573	3.8992944
C	-1.1849282	1.3437221	4.3801898
H	-1.6729662	2.1771142	3.8958615
C	0.1882887	1.0359465	4.3347251
H	0.9573403	1.5914956	3.8194628
C	0.4017245	-0.1433723	5.0796970
H	1.3562559	-0.6306730	5.2200076
C	-0.8412147	-0.5611698	5.5923854
H	-1.0142187	-1.4369102	6.2017430
C	-1.8250934	0.3503395	5.1513274
Y	2.2343632	-2.0667905	-1.1743770
C	2.5400161	-4.7300861	-1.4061121
H	3.5555944	-5.0940028	-1.3348505

C	1.8851653	-4.3178453	-2.5897176
H	2.3065057	-4.3182447	-3.5857044
C	0.5807820	-3.9120612	-2.2452705
H	-0.1607919	-3.5230574	-2.9261112
C	0.4363149	-4.0524296	-0.8524562
H	-0.4401621	-3.8138061	-0.2697996
C	1.6469384	-4.5615743	-0.3343882
C	4.8797325	-2.2416460	-1.0118566
C	4.5913213	-0.9264809	-0.5890769
H	4.7014508	-0.5507431	0.4177730
C	4.1125993	-0.2106247	-1.7028776
H	3.7941480	0.8207844	-1.7186086
C	4.1075066	-1.0776822	-2.8162789
H	3.7829241	-0.8141039	-3.8130769
C	4.5917167	-2.3294638	-2.3910261
Y	-2.4940874	0.3733611	-2.0671739
As	-2.2547177	0.6777989	0.8285742
As	1.4992673	-1.2661349	1.5517347
As	0.3574266	-0.2157515	-2.4351542
C	-3.9750227	-1.7680515	-1.3518631
H	-4.2031071	-1.8366457	-0.2992167
C	-2.8413758	-2.3020084	-1.9922126
H	-2.0514082	-2.8734834	-1.5297516
C	-2.9067513	-1.9581641	-3.3599686
H	-2.1702619	-2.2091535	-4.1092586
C	-4.0777956	-1.2081003	-3.5627537
H	-4.4075845	-0.7876733	-4.5025639
C	-4.7356286	-1.0798090	-2.3177284
C	-1.9282165	2.6096637	-3.4370610
H	-0.9214985	2.6897677	-3.8208089
C	-2.3855440	3.0613505	-2.1843923
H	-1.8020907	3.5637047	-1.4276404
C	-3.7547074	2.7387845	-2.0714549
H	-4.3794152	2.9529903	-1.2157480
C	-4.1456233	2.0924455	-3.2595010
H	-5.1339195	1.7151908	-3.4809114
C	-3.0151416	2.0013154	-4.1001689
C	-2.8824256	2.4512099	1.3989764
C	-2.0308461	3.5636721	1.5610306
C	-2.5461805	4.7951125	1.9600635
H	-1.8665695	5.6352927	2.0744876
C	-3.8964593	4.9618415	2.2135012
C	-4.7401335	3.8731916	2.0692689
H	-5.8025599	3.9827444	2.2713837
C	-4.2581507	2.6290504	1.6703126
C	-0.5576457	3.4515598	1.3302377
H	-0.1301209	2.6760511	1.9706249
H	-0.0503531	4.3981223	1.5341455
H	-0.3401738	3.1544023	0.3006862
C	-5.2321512	1.4967405	1.5470633

H	-5.2519118	1.0990291	0.5281396
H	-6.2393097	1.8203026	1.8267454
H	-4.9329116	0.6559539	2.1794897
C	2.9991485	-0.5946766	2.6305317
C	3.6011351	-1.4623650	3.5700031
C	4.6613807	-1.0190130	4.3565009
H	5.1057798	-1.7063738	5.0717957
C	5.1561674	0.2692663	4.2414687
C	4.5808718	1.1226967	3.3162936
H	4.9598804	2.1350970	3.2054485
C	3.5190475	0.7111669	2.5135225
C	3.1307219	-2.8746812	3.7436686
H	2.0800662	-2.9092004	4.0465872
H	3.7376416	-3.3957901	4.4902575
H	3.1757947	-3.4188053	2.7958690
C	2.9622824	1.6809938	1.5206181
H	2.9880970	1.2548161	0.5145159
H	3.5239768	2.6187479	1.5235144
H	1.9133175	1.9049446	1.7327064
C	1.2807410	0.9657719	-3.7059676
C	1.4910833	0.5094052	-5.0271881
C	2.1525354	1.3141984	-5.9512930
H	2.3022590	0.9384095	-6.9602486
C	2.6163077	2.5736310	-5.6101187
C	2.4040847	3.0342979	-4.3225030
H	2.7534308	4.0230192	-4.0374752
C	1.7449289	2.2555744	-3.3734829
C	1.0094519	-0.8370486	-5.4754757
H	1.4650980	-1.6386233	-4.8866263
H	1.2346450	-0.9960102	-6.5343780
H	-0.0678629	-0.9374086	-5.3147839
C	1.5319155	2.8297777	-2.0090275
H	0.4686138	2.8332080	-1.7569845
H	1.9144569	3.8518257	-1.9452421
H	2.0250949	2.2247185	-1.2435145
Li	0.2457796	0.4966112	0.0734526
H	5.2585224	-3.0376569	-0.3852609
H	5.9824689	0.6019660	4.8616173
H	3.1320387	3.1897312	-6.3398467
H	-4.2858672	5.9263637	2.5235113
H	4.7049655	-3.2090264	-3.0089072
H	1.8531960	-4.7593798	0.7073110
H	-5.6621756	-0.5523624	-2.1364779
H	-2.9826921	1.5493150	-5.0821819
H	-0.8894861	-3.7074278	4.7347683
H	-2.8839170	0.3027123	5.3665912

References

1. a) G. Wilkinson, J. M. Birmingham, *J. Am. Chem. Soc.*, **1954**, *76*, 6210. b) J. Darkwa, D. M. Giolando, C. Jones Murphy, T. B. Rauchfuss, *Inorg. Synth*. **1990**, *27*, 51.
2. K. Izod, S. T. Liddle, C. William, *Inorg. Chem.* **2004**, *43*, 214.
3. A. J. Roering, J. J. Davidson, S. N. MacMillan, J. M. Tanski, R. Waterman, *Dalton Trans*. 2008, 4488.
4. O. V. Dolomanov, L. J. Bourhis, R. J. Gildea, J. A. K. Howard, H. Puschmann, *J. Appl. Cryst*. **2009**, *42*, 339.
5. R. Ahlrichs, M. Bär, M. Häser, H. Horn, and C. Kölmel, *Chem. Phys. Lett.*, **1989**, *162*, 165.
6. J. Perdew, K. Burke, and M. Ernzerhof, *Phys. Rev. Lett.*, **1996**, *77*, 3865.
7. C. Adamo and V. Barone, *J. Chem. Phys.*, **1999**, *110*, 6158.
8. F. Weigend and R. Ahlrichs, *Phys. Chem. Chem. Phys.*, **2005**, *7*, 3297.
9. A. Schäfer, C. Huber, and R. Ahlrichs, *J. Chem. Phys*., **1994**, *100*, 5829.
10. A. E. Reed, R. B. Weinstock, and F. Weinhold, *J. Chem. Phys.*, **1985**, *83*, 735.
11. R. F. W. Bader, *Atoms in Molecules: A Quantum Theory*, Oxford University Press, Oxford, 1990.
12. E. D. Glendening, J. K. Badenhoop, E. A. Reed, J. E. Carpenter, J. A. Bohmann, C. M. Morales, C. R. Landis, and F. Weinhold, **2013**, *NBO 6.0 , Theor. Chem. Institute, Univ. Wisconsin, Madison, WI.*
13. T. A. Keith, **2014**, *AIMAll (Version 14.11.23), TK Gristmill Software, Overl. Park KS, USA.*
